# Identification of Serum Exosomal hsa-circ-0004771 as a Novel Diagnostic Biomarker of Colorectal Cancer

**DOI:** 10.3389/fgene.2019.01096

**Published:** 2019-11-01

**Authors:** Bei Pan, Jian Qin, Xiangxiang Liu, Bangshun He, Xuhong Wang, Yuqin Pan, Huiling Sun, Tao Xu, Mu Xu, Xiaoxiang Chen, Xueni Xu, Kaixuan Zeng, Li Sun, Shukui Wang

**Affiliations:** ^1^General Clinical Research Center, Nanjing First Hospital, Nanjing Medical University, Nanjing, China; ^2^School of Medicine, Southeast University, Nanjing, China; ^3^Department of Laboratory Medicine, The Second Affiliated Hospital of Nanjing Medical University, Nanjing, China

**Keywords:** hsa-circ-0004771, circular RNA, exosome, colorectal cancer, diagnosis, biomarker

## Abstract

**Background:** Exosomal circular RNAs (circRNAs) in peripheral blood are considered as emerging diagnostic biomarkers of cancers. Owing to the lack of sensitive and specific biomarkers, a large number of colorectal cancer (CRC) patients were diagnosed in advanced stages leading to high mortality. This study aimed to identify circulating exosomal circRNAs as novel diagnostic biomarkers of CRC.

**Materials and Methods:** Candidate circRNA was selected by integrating analysis of Gene Expression Omnibus (GEO) database with online program GEO2R. A total of 170 patients and 45 healthy controls were enrolled to assess the diagnostic value of circRNAs for CRC. Exosomes isolated from the serum of participants and cell cultured media were confirmed by transmission electron microscope (TEM), Nanoparticle Tracking Analysis and western blot. The expression and the diagnostic utility of circRNA were tested by qRT-PCR and receiver operating characteristic (ROC) analysis, respectively.

**Results:** The circulating exosomal hsa-circ-0004771 with most abundant among the top ten differentially expressed circRNAs (fold change ≥1.5) was selected for further study based on the results of GEO dataset analysis. The up-regulated exosomal hsa-circ-0004771 was verified in serum of CRC patients compared to healthy controls (HCs) and patients with benign intestinal diseases (BIDs) by qRT-PCR. The area under the ROC curves (AUCs) of circulating exosomal hsa-circ-0004771 were 0.59 (95%CI, 0.457–0.725), 0.86 (95%CI, 0.785–0.933) and 0.88 (95%CI, 0.815–0.940) to differentiate BIDs, stage I/II CRC patients and CRC patients from HCs, respectively. The AUC was 0.816 (95%CI, 0.728–0.9) to differentiate stage I/II CRC patients from patients with BIDs. In addition, the elevated expression of exosomal hsa-circ-0004771 in the serum of CRC patients was tumor-derived. It was found that the expression of exosomal hsa-circ-0004771 was down-regulated expression of in the serum of postoperative CRC patients as well as cultured media of CRC cells treated with GW4869.

**Conclusions:** Circulating exosomal hsa-circ-0004771 was significantly up-regulated in CRC patients and served as a novel potential diagnostic biomarker of CRC.

## Introduction

Colorectal cancer (CRC) is the third most common cancer and the second leading cause of death globally (Bray et al., 2018). Clinical screenings for CRC include fecal occult blood test, colonoscopy screening and conventional tumor biomarkers (Levin et al., 2003; Cho, 2011). However, almost 90% of deaths might be preventable if CRC patients were diagnosed at an early stage (Smith et al., 2001). Therefore, sensitive diagnostic biomarkers for early detection of CRC are urgently needed.

Exosomes are extracellular vesicles, which contain multiple proteins, lipids, DNA and different RNA species (Pan and Johnstone, 1983). Increasing findings have indicated that exosome-delivered RNAs involve in the occurrence and progress of cancers, which also involved in epithelial–mesenchymal transition (Wang et al., 2014), angiogenesis (Umezu et al., 2014), premetastatic niche (Grange et al., 2011), immune response and therapeutic resistance (Xie et al., 2019). Furthermore, isolation of exosomes from accessible bodily fluids (blood, urine, saliva, etc.) made it possible to apply exosomes in clinical diagnosis (Skog et al., 2008; Oeyen et al., 2018; Usman et al., 2018; Vo et al., 2019). Some exosome-delivered circRNAs, such as circPTGR1, have been reported that it is stably present in exosomes and play an essential role in hepatocellular carcinoma development (Wang et al., 2019; Zhang et al., 2019a).

Circular RNAs (circRNAs) are a kind of single-stranded covalently closed RNAs (Chen et al., 2019a), which were initially identified as splicing-associated noise in the early 1990s and formed by backsplicing process of pre-mRNA (Capel et al., 1993; Conn et al., 2015). Recently, a number of studies reported that circRNAs were closely associated with the initiation and development of cancers, including CRC (Li et al., 2018; de Fraipont et al., 2019; Jin et al., 2019). In fact, circRNAs are more stable than linear RNAs due to their special loop structure which lack of 5’-3’ polarity and polyadenylate tail. This could prevent from RNase R degradation or RNA exonuclease digestion (Suzuki and Tsukahara, 2014). Hence, stable circRNAs are rich in peripheral blood and has potential as biomarkers of diseases (Chen et al., 2017; Wang et al., 2019).

In this study, we screened exosomal hsa-circ-0004771 as a candidate for potential diagnostic biomarker of CRC by database analysis, and explored its expression in exosome of sera, CRC tissues and cells. Our results revealed that exosomal hsa-circ-0004771 was significantly up-regulated in sera of CRC patients with significant diagnostic efficacy, it could offer new opportunities for potential diagnosis targeting colorectal cancer.

## Materials and Methods

### Study Population

A total of 135 CRC patients, 35 patients with benign intestinal diseases (BIDs) and 45 healthy controls (HCs) were enrolled from Nanjing First Hospital affiliated to Nanjing Medical University. CRC patients were confirmed by histopathological analysis of the surgical resection tissues. All pre-operation serum samples were collected from patients with treatment naïve. The HCs were recruited from disease-free healthy volunteers and subjects for body check. Written informed consents were obtained from each participant, and the study protocol was approved by the Research and Ethical Committee of Nanjing First Hospital. The information about clinicopathological characteristics of all participants was retrieved from medical records and questionnaires, which was summarized in **Table 1**.

**Table 1:** Clinical characteristics of the participants.

Parameters	Discovery Phase		Validation Phase			Exploration Phase	
	HC (n = 10)	CRC (n = 10)	HC (n = 35)	BID (n = 35)	CRC (n = 110)	CRC Tissues and ANTs (n = 5)	Pre/Post-Operative (n = 10)
**Age, years (mean ± SD)**	58 ± 7.6	62 ± 9.3	60 ± 10.8	56 ± 10.2	60 ± 13.3	59 ± 8.7	61 ± 4.8
**Gender (M/F)**	5/5	5/5	19/16	21/14	76/34	4/1	8/2
**Drinking**	4	5	17	27	71	5	6
**BID**							
**Acute/Chronic Enteritis**	\	\	\	19	\	\	\
**Intestinal Polyps**	\	\	\	6	\	\	\
**Intestinal Obstruction**	\	\	\	8	\	\	\
**Crohn’s Disease**	\	\	\	2	\	\	\
**TNM stage**							
**I**	\	2	\	\	29	2	6
**II**	\	4	\	\	41	3	3
**III**	\	3	\	\	27	\	1
**IV**	\	1	\	\	13	\	0

CRC, colorectal cancer; HC, healthy control; BID, benign intestinal diseases; ANT, adjacent normal tissue; M, male; F, female.

### Study Design

**Figure 1:**
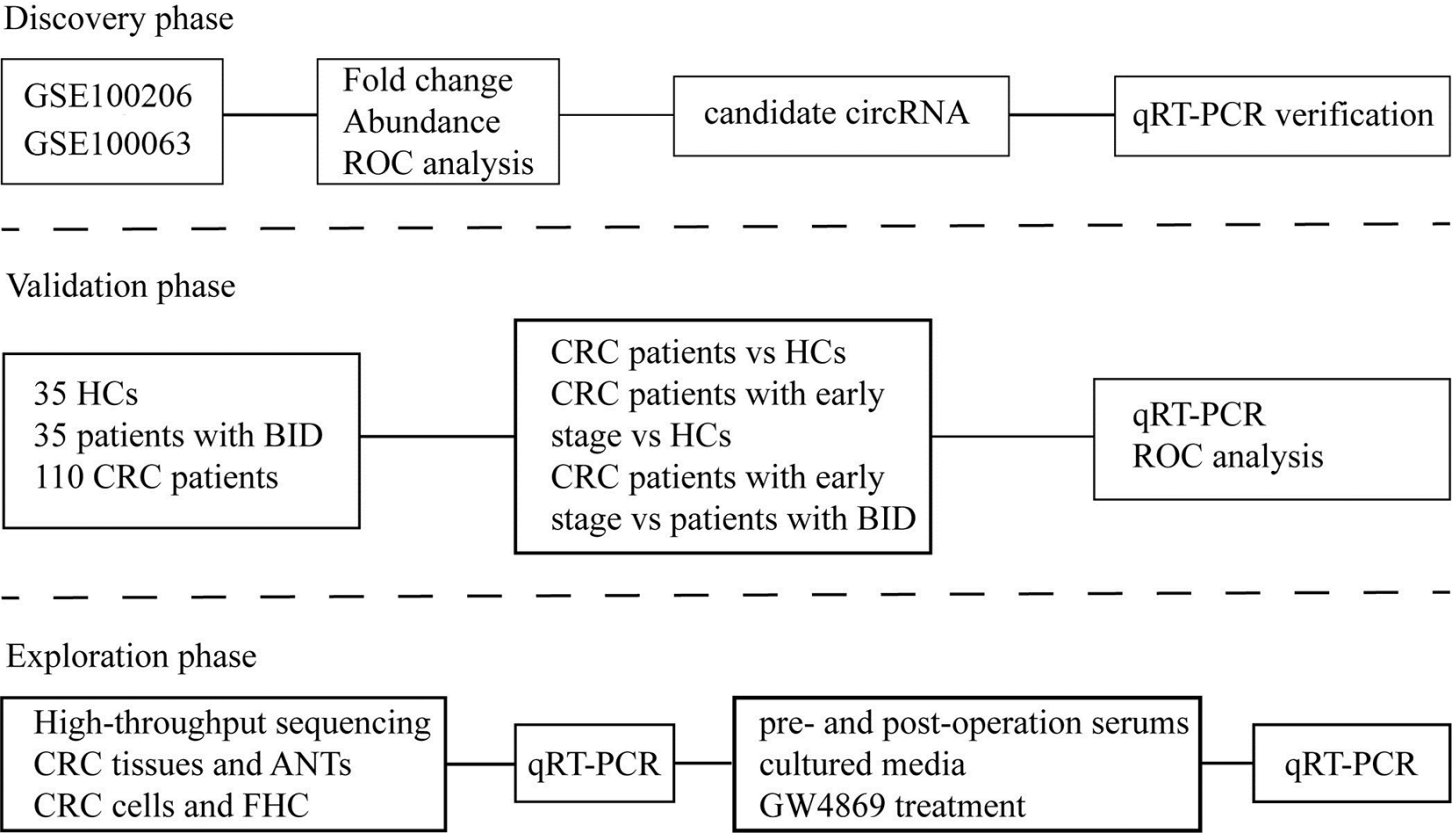
Flow chart of the study design. ROC, receiver operating characteristic; qRT-PCR, quantitative real-time polymerase chain reaction; HC, healthy control; BID, benign intestinal diseases; CRC, colorectal cancer; ANT, adjacent normal tissue.

As shown in **Figure 1**, our study was conducted in three phases, discovery phase, validation phase, and exploration phase. In the discovery phase, we selected exosomal hsa-circ-0004771 as a target gene of CRC based on the integrated analysis of two Gene Expression Omnibus (GEO) datasets and verification in the age-matched HCs and BC patients by using qRT-PCR. In the validation phase, the diagnostic value of exosomal hsa-circ-0004771 was assessed by its relative expressions among 180 subjects, including 70 stage I/II CRC patients, 40 stage III/IV CRC patients, 35 patients with BIDs, and 35 HCs). Finally, in the exploration phase, the origin of exosomal hsa-circ-0004771 in serum of CRC patients was explored in tumor tissue and CRC cell lines.

### Cell Culture

Human colorectal mucosal epithelial cell (FHC) and CRC cell lines (HCT-116 and SW-480) were obtained from American Type Culture Collection. FHC, HCT-8 and HCT-116 cells were cultured in RPMI-1640 with 10% fetal bovine serum (FBS) and other cells were maintained in Dulbecco’s Modified Eagle’s Medium (DMEM). All of the cell lines are bathed with 10% FBS and 1% penicillin/streptomycin (P/S) solution and incubated in an incubator containing 5% CO_2_ at 37°C.

### Isolation of Exosomes

Exosomes were isolated from serum and medium samples using Invitrogen^™^ Total Exosome Isolation Kits (4478359 and 4478360) (Invitrogen, NYC, USA) according to the manufacturer’s protocol. For serum samples, serum was collected from venous blood after centrifugation at 2,000 × *g* for 20 min and subsequently mixed with the exosome isolation reagent. The mixture was centrifuged at 15,000 × *g*, 4°C for 2 min after standing for 30 min, then the supernatant was removed to obtain exosome precipitation. For cell lines, the culture media were collected for exosome isolation from culture plates with cells being cultivated for 24 h or 48 h, and then the exosomes were isolated according to the manufacturer’s protocol.

### Extraction of RNAs and qRT-PCR

Total RNA was extracted from exosomes, cells and tissues respectively, by using TRIzol reagent (Invitrogen, NYC, USA). Thereafter, 1 ug total RNA was added to a final volume of 20 µl mixed reagent for reverse transcription. Then different expression of hsa-circ-0004771 from exosomes, cells and tissues real-time PCR (qRT-PCR) was conducted in triplicate with specific primers of hsa-circRNAs, which were chemically synthesized and validated in RiboBio Company (RiboBio, Guangdong, China). The sequence of circ-00047771: former primer, AGTTGCTCCAATGACAGAGTTACC; reverse primer,CCTCCTTCAGTCAAGTGTGCATC.

The PCR was conducted on ABI 7500 real-time PCR system (Applied Biosystems, CA, USA) used SYBR Green (Takara, China) The relative expression levels of circRNAs were calculated by the 2^^–ΔΔCt^ method.

### Transmission Electron Microscopy Analysis

Isolated exosomes were re-suspended in 100 µl phosphate-buffered saline (pH 7.4) and fixed in 50 µl glutaraldehyde. Subsequently, fixed exosomes were dropped onto a formvar-carbon coated grid, left to dry at room temperature for 5 min, excess liquid was removed and then stained with phosphotungstic acidoxalate for 1 min. Then, the grid was further dried at room temperature for 10 min and visualized on Tecnai G2 F20 transmission electron microscope (TEM) (FEI, United States) at 185 kV.

### Western Blot

To test the expression of exosome specific protein CD63 and TSG101, the whole proteins were extracted from serum of subjects, and culture media of cell lines by using the Whole protein extraction kit (KGP2100). The protein concentration was measured by bicinchoninic acid (BCA) kit (KGPBCA) (KeyGEN BioTECH, China). After immunoblotting, the proteins attached to polyvinylidene fluoride (PVDF) membrane were incubated overnight at 4°C with specific antibodies, and subsequently incubated with HPR-labeled secondary antibodies. Finally, the membrane was exposed with chemiluminescent agents.

### Bioinformatics Analysis

We searched the GEO database with the following keywords restricting, namely (“circRNA” OR “circRNAs” OR “Circular RNAs”), (“colorectal carcinoma” OR “colorectal cancer” OR “colorectal neoplasm” OR “colorectal tumor” OR “CRC”) and (“exosome” OR “exosomes”). The differentially expressed circRNAs in GEO datasets were analyzed by using online tool GEO2R.

### Statistical Analyses

Chi-square test was used to compare the clinicopathological features of each group and the Student’s t test was conducted to examine differences in expression of circRNAs. Receiver operating characteristic (ROC) curve and the area under the ROC curve (AUC) were constructed to assess the diagnostic performance of hsa-circ-0004771, and the corresponding cutoff points of the ROC curve were determined by the Youden’s Index. The sensitivity, specificity and 95% confidence intervals (CIs) were calculated using the binary regression model (Deville et al., 2002). All of the data were analyzed by IBM SPSS version 19.0 (IBM Corp, NYC, USA) and the graphs were generated by GraphPad Prism 8 software (GraphPad Software, CA, USA) and R language. The P value < 0.05 is considered statistically significant.

## Results

### Exosomal hsa-circ-0004771 Was Significantly Up-Regulated in Sera of CRC Patients With Remarkable Diagnostic Value

**Figure 2:**
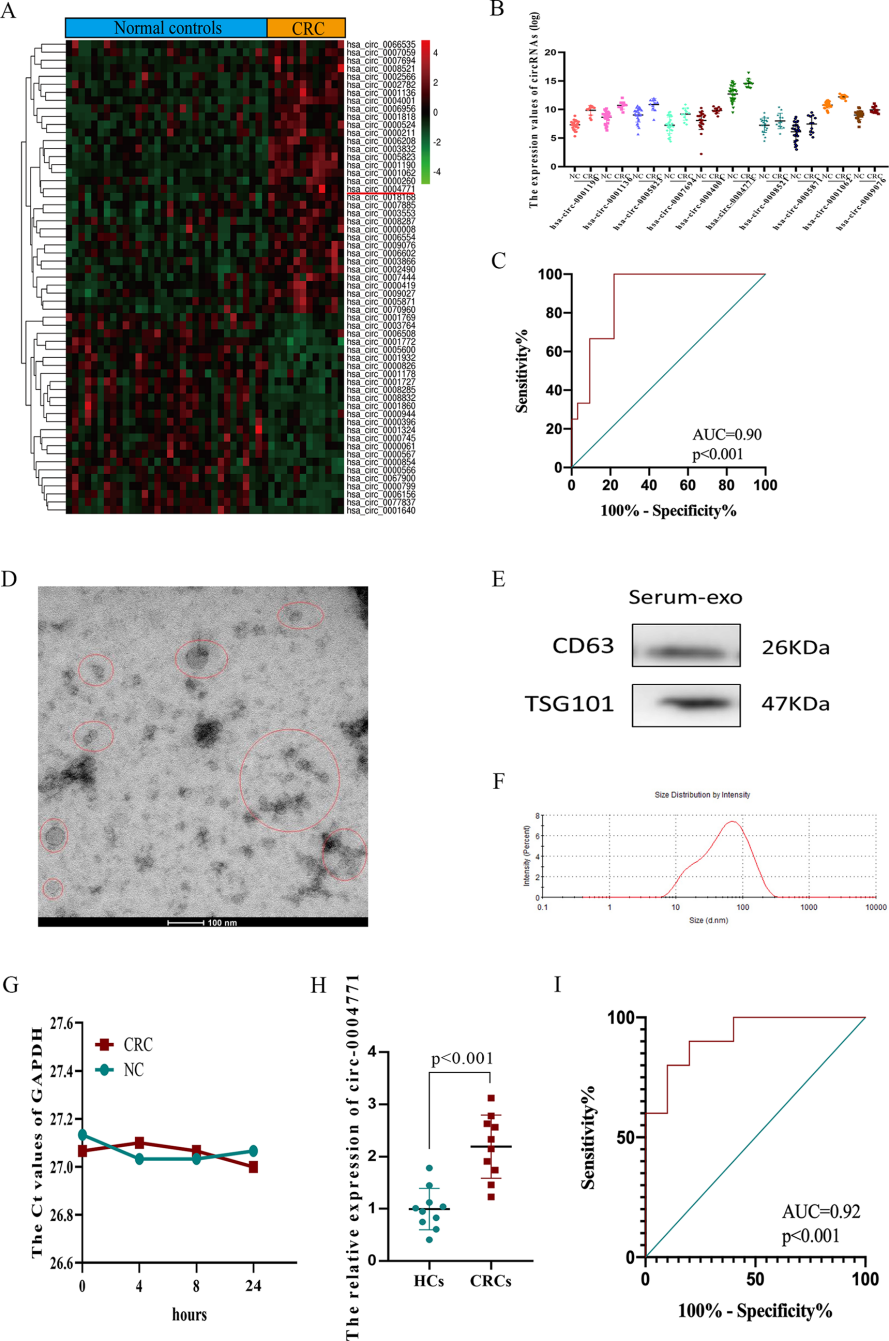
Identification and validation of up-regulated exosomal hsa-circ-0004771 in peripheral blood of CRC patients.**(A)** Heat maps of DEcircRNAs of analysis of GSE100206 and GSE100063. **(B)** Expression of DEcircRNAs in GEO datasets. **(C)** ROC analysis of hsa-circ-0004771 in GEO datasets (*P* < 0.001). **(D**–**F)** TEM, western blot analysis and nanosight particle tracking of peripheral blood exosomes. **(G)** The expression value of exosomal GAPDH in serum of CRC patients and HCs at different standing times. **(H)** qRT-PCR analysis of hsa-circ-0004771 expressions in peripheral blood exosomes of CRC patients (n = 10) and HCs (n = 10). **(I)** ROC analysis of circulating exosomal hsa-circ-0004771 to differentiate CRC patients from HCs (*P* < 0.001). DEcircRNAs, differentially expressed circRNAs; ROC, receiver operating characteristic; TEM, transmission electron microscope; qRT-PCR, quantitative real-time polymerase chain reaction; CRC, colorectal cancer; HC, healthy control.

After analysis of difference and abundance expression of circRNAs in GSE100206 (n = 32) and GSE100063 (n = 12) datasets, 59 circulating exosomal circRNAs with fold change ≥1.5 and P value <0.05 were identified as differentially expressed circRNAs (DEcircRNAs) (**Figure 2A**). We selected hsa-circ-0004771 for the study as the expression of hsa-circ-0004771 was the most abundant among top 10 up-regulated ones (**Figure 2B**, **Supplement Table 1**). ROC analysis showed that the diagnostic value of circulating exosomal hsa-circ-0004771 to differentiate CRC patients from HCs was remarkably high (AUC = 0.90) (**Figure 2C**). To further confirm the results, we enrolled 10 CRC patients and 10 healthy controls, and the isolated exosomes from clinical serum samples were verified through TEM (**Figure 2D**), western blot (**Figure 2E**) and nanosight particle tracking analysis (**Figure 2F**). GAPDH was used as an internal reference of exosomal circRNAs in peripheral blood because the expression of exosomal GAPDH in serum had no difference between CRC patients and HCs and did not obviously change during the incubation time (4, 8, 24 h) at room temperatures (**Figure 2G**). Up-regulated expression was consistently observed in serum of CRC patients compared to HCs by qRT-PCR (**Figure 2H**). ROC analysis showed that the AUC of circulating exosomal hsa-circ-0004771 was 0.92 to differentiate CRC patients from HCs (**Figure 2I**).

### Validating the Expression and Diagnostic Value of Circulating Exosomal hsa-circ-0004771 in an Independent Cohort

**Figure 3:**
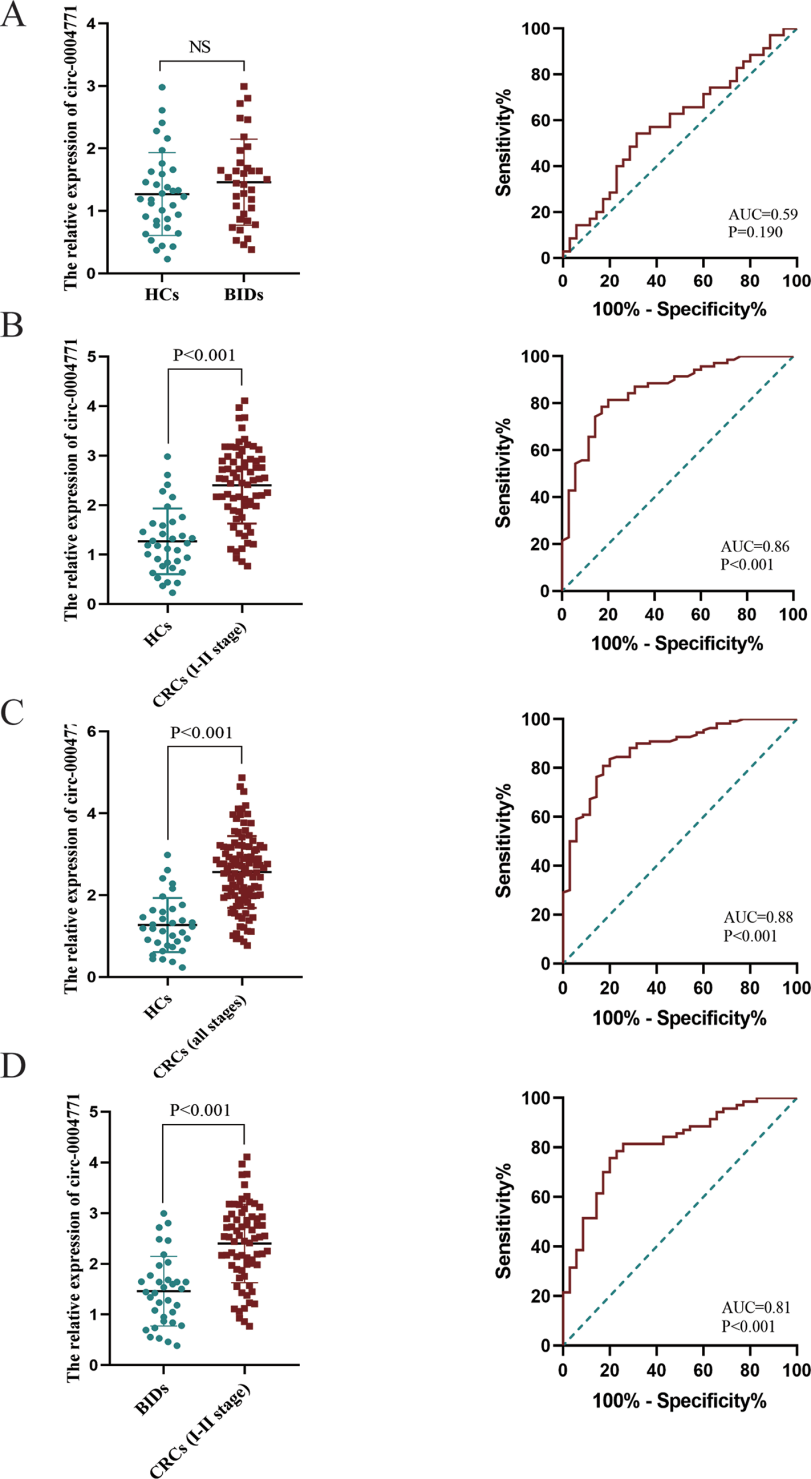
Dysregulated circulating exosomal hsa-circ-0004771 serves as a promising diagnostic biomarker for CRC. **(A**–**C)** qRT-PCR and AUC analysis of hsa-circ-0004771 expression in patients with BID (n = 35), stage I/II CRC patients (n = 70) and CRC patients (all stage) (n = 110) compared with HCs (n = 35), respectively. **(D)** qRT-PCR and AUC analysis of hsa-circ-0004771 expression in patients with BID (n = 35) compared with stage I/II CRC patients (*P* < 0.001). qRT-PCR, quantitative real-time polymerase chain reaction; CRC, colorectal cancer; HC, healthy control; BID, benign intestinal diseases; ROC, receiver operating characteristic.

To validate the dysregulated expression of circulating exosomal hsa-circ-0004771, serum samples of 110 CRC patients, 35 patients with BID, and 35 HCs, were tested. First, we observed that there was no significant difference in the expression of serum exosomal circ-0004771 between HCs and CRC patients, and the corresponding AUC was 0.59 with the sensitivity of 54.29% and specificity of 68.57% (*P* = 0.190) (**Figure 3A**). Then, we compared the expression circulating exosomal hsa-circ-0004771 between stage I/II CRC patients and HCs. As shown in **Figure 3B**, exosomal hsa-circ-0004771 was also significantly elevated in the serum of stage I/II CRC patients. The AUC was 0.86 (95%CI, 0.785–0.933) to discriminate stage I/II CRC patients from HCs with the sensitivity of 81.43% and specificity of 80% (*P* < 0.001). When compared with HCs, the expression of exosomal hsa-circ-0004771 in serum was significantly up-regulated in CRC patients with all TNM stages. The AUC of circulating exosomal hsa-circ-0004771 to differentiate CRC patients with all stages from HCs was 0.88 (95%CI, 0.815–0.940) with the sensitivity of 80.91% and specificity of 82.86% (*P* < 0.001) (**Figure 3C**). Subsequently, we found that the expressions of exosomal hsa-circ-0004771 in serum were significantly up-regulated in stage I/II CRC patients to differentiate patients with BIDs from stage I/II CRC patients was 0.81 (95%CI, 0.728–0.900) with the sensitivity of 81.43% and specificity of 74.29% (*P* < 0.001) (**Figure 3D**). Finally, the Chi-square test revealed that expression of circulating exosomal hsa-circ-0004771 was significantly correlated with TNM stage (*P* = 0.017) and distant metastasis (*P* = 0.004) (**Table 2**).

**Table 2:** Correlations between hsa-circ-0004771 expression and clinicopathological features of CRC patients in serum exosomes.

Characteristics	Number of Cases	Expression of hsa-circ-0004771		P value^a^
		Low (n = 55)	High (n = 55)	
**Age, years**	110			
**< 50**	36	20	16	0.416
**≥50**	74	35	39	
**Gender**				
**Male**	72	38	34	0.423
**Female**	38	17	21	
**TNM stage**				
**I–II**	70	41	29	**0.017**
**III–IV**	40	14	26	
**Lymphatic Metastasis**				
**N0**	87	46	41	0.241
**N1 + N2**	23	9	14	
**Distant Metastasis**				
**M0**	88	50	38	**0.004**
**M1**	22	5	17	

a Statistical significant results (in bold) (P < 0.05).

### Elevated Circulating Exosomal hsa-circ-0004771 in CRC Patients was Tumor-Derived

**Figure 4:**
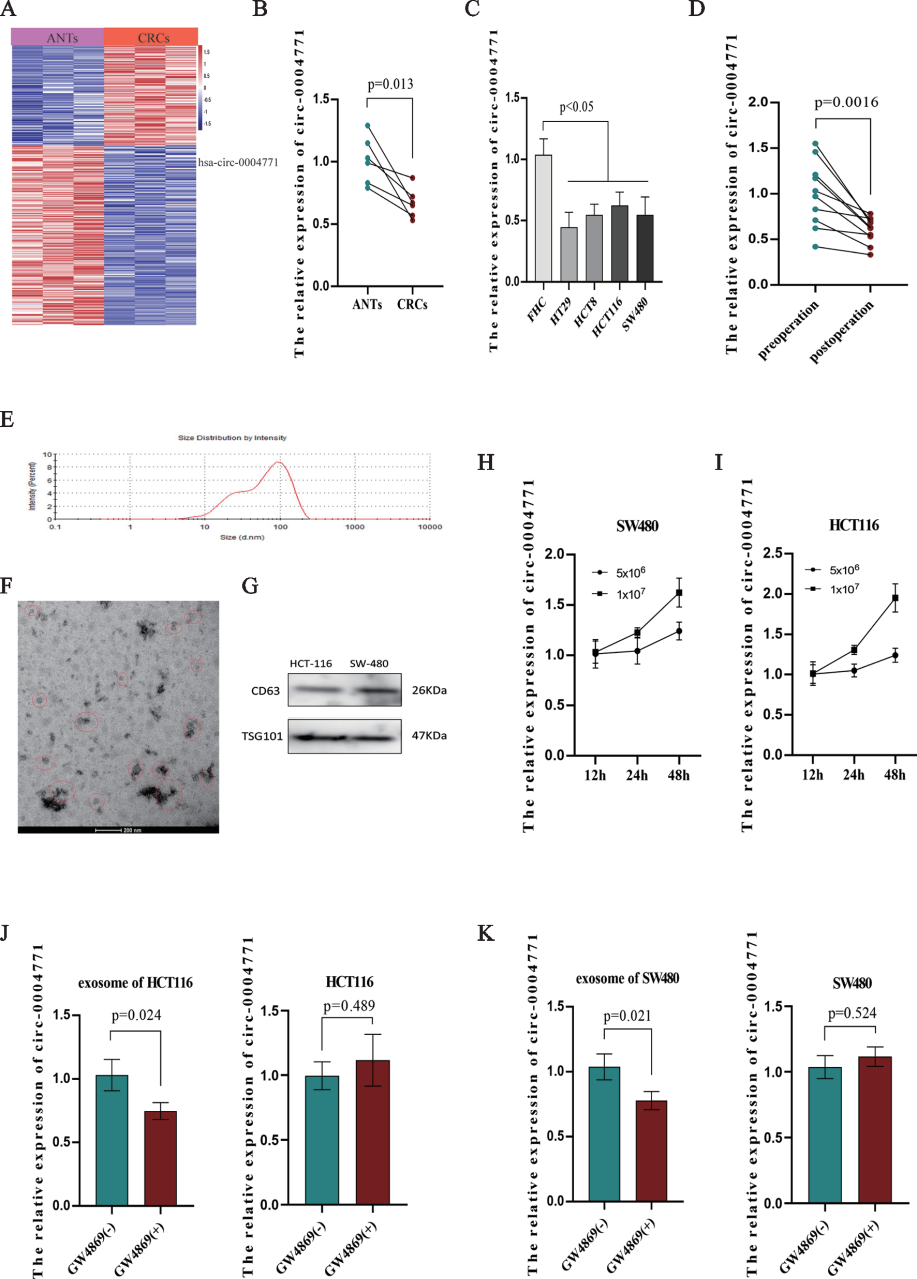
Elevated expression of circulating exosomal hsa-circ-0004771 was tumor-derived. **(A)** Heat map of differentially expressed circRNAs in CRC tissues and ANTs by high-throughput sequencing. Red in the two plots denotes up-regulation; blue denotes down-regulation. **(B)** qRT-PCR analysis of hsa-circ-0004771 expression in five pairs of CRC tissues and ANTs. **(C)** qRT-PCR analysis of hsa-circ-0004771 expression in colorectal mucosal epithelial cell (FHC) and CRC cells. **(D)** qRT-PCR analysis of hsa-circ-0004771 expression in 10 pairs of preoperative serum samples and corresponding postoperative samples. **(E**–**G)** TEM, and western blot analysis nanosight particle tracking of exosomes in cell cultured media (HCT-116 and SW-480). **(H** and **I)** qRT-PCR analysis of hsa-circ-0004771 expression in cultured media with different time points and cell numbers. **(J** and **K)** qRT-PCR analysis of hsa-circ-0004771 expression in CRC cells and corresponding cultured media after treated with GW4869. CRC, colorectal cancer; ANT, adjacent normal tissue; qRT-PCR, quantitative real-time polymerase chain reaction; TEM, transmission electron microscope.

Several studies reported cancer cells may release circRNAs in the cargo of exosomes into the peripheral blood (Dou et al., 2016; Imaoka et al., 2016; Lasda and Parker, 2016). To explore the source of up-regulated exosomal hsa-circ-0004771, we firstly examined the results of high-throughput sequencing using three paired CRC tissues and adjacent normal tissues (ANTs). Unexpectedly, we found that hsa-circ-0004771 was significantly down-regulated in CRC tissues (**Figure 4A**), and qRT-PCR verified hsa-circ-0004771 was significantly down-regulated in CRC tissues and cells (**Figures 4B–C**). To explore the origin of circulating exosomal hsa-circ-0004771, we firstly examined ten pairs of serum specimens collected from CRC patients before and after surgery operation. The result shown that the expression of circulating exosomal hsa-circ-0004771 was significantly decreased after operation (**Figure 4D**), suggesting increased expression of exosomal hsa-circ-0004771 may be tumor-derived. Meanwhile, the results of relative expression of exosomal hsa-circ-0004771, which were also verified by TEM, nanosight particle tracking analysis and western blot (**Figures 4E–G**). In cultured media of CRC cells (HCT-116 and SW-480) revealed that the increased exosomal hsa-circ-0004771 expression was depended on the cell numbers and culture time (**Figures 4H–I**). In addition, we applied GW4869, a known blocker for exosomes, to inhibit the exosomes secretion. We found that the expression of exosomal hsa-circ-0004771 was significantly down-regulated in cultured media, but not significantly changed in the CRC cells (**Figures 4J–K**).

## Discussion

In this study, we first sought to determine circulating exosomal hsa-circ-0004771 was significantly up-regulated in the sera of CRC patients and showed a high diagnostic value for CRC patients. Subsequently, elevated exosomal hsa-circ-0004771 in serum of CRC patients was demonstrated to be tumor-derived.

With the advent of bioinformatics analysis and high-throughput sequencing technology, functional circRNAs have been extensively elucidated. CircRNAs are stable and abundant in cells due to their special covalently closed cyclic structure. They also being reported as key regulators in biological progression of cancers (Beermann et al., 2016). Up to now, rising evidence reveals that circRNAs are closely associated with angiogenesis, tumor microenvironment and metastasis of various cancers (Zhong et al., 2017; Chen et al., 2019b; Karedath et al., 2019). For example, Bahn et al. reported that in human saliva, 422 circRNAs might participate in inflammatory and chemotaxis responses and regulate microenvironment of cancer (Bahn et al., 2015). In CRC, circCCDC66 sponged miRNA-33b and miR-93 to protect the MYC mRNA so that tumor proliferation, migration, and metastasis were activated in both *in vitro* and *in vivo* (Hsiao et al., 2017). However, the role of hsa-circ-0004771 (circNRIP1) and its diagnostic value in cancer remain poorly characterized. So far, hsa-circ-0004771 was only shown as an oncogene in gastric cancer (GC), knockdown of hsa-circ-0004771 blocked malignant tumor phenotype of GC cells (Zhang et al., 2019b). Our study revealed that exosomal hsa-circ-0004771 was significantly up-regulated in sera of CRC patients and the increased expression of exosomal hsa-circ-0004771 may be tumor-derived. However, the biological role of hsa-circ-0004771 in CRC still needed further investigation.

In recent years, exosome have been widely studied as an important regulatory node for the intercellular interaction of cells (Chan et al., 2019; Hu and Li, 2019), and the exosomes derived from tumor cells could package circRNAs and be released into the circulation system (Bach et al., 2019). Moreover, circulating circRNAs in exosome as biomarkers of diseases has aroused great attention (Fang et al., 2018; Ma et al., 2019). We discovered exosomal hsa-circ-0004771 was significantly up-regulated in the sera of CRC patients and identified as a novel potential biomarker for the early diagnosis of CRC patients based on the public data uploaded into the GEO database. Interestingly, we found hsa-circ-0004771 was down-regulated in exosome of CRC cells and CRC tissues, which may be consistent with the idea of Lasda et al. who supposed that the active transport of circRNAs to exosomes may be a mechanism for circRNAs clearance (Lasda and Parker, 2016). CircRNAs, which are enriched and stable in cells, may be bound to RNA binding proteins and transported to exosomes (Dou et al., 2016; Zang et al., 2018), but the actual sorting mechanism of circRNAs into exosomes needed to be further investigated.

In conclusion, we demonstrated that the exosomal hsa-circ-0004771 was significantly up-regulated in CRC patients’ sera. Circulating exosomal hsa-circ-0004771 could serve as a novel potential biomarker for early diagnosis of CRC.

## Data Availability Statement

The datasets analyzed for this study can be found in the Gene Expression Omnibus, accession numbers GSE100206 and GSE100063. The original qPCR data generated for this manuscript is available on request to the corresponding author.

## Ethics Statement

The informed consents were signed by each participant, and study protocol was approved by the Research and Ethical Committee of Nanjing First Hospital.

## Author Contributions

The research project was designed by BP and SW, organized by BH, and statistical analysis was designed by JQ, XW, HS, TX, LS, YP, MX, XC, and KZ. The first draft of the manuscript was written by BP and JQ, and the manuscript was reviewed and critiqued by XX, XL, and BH.

## Funding

This project was supported by grants from Key Project of Science and Technology Development of Nanjing Medicine (ZDX16001) to SW; The National Nature Science Foundation of China (No. 81802093) to HS; Innovation team of Jiangsu provincial health-strengthening engineering by science and education (CXTDB2017008); Jiangsu Youth Medical Talents Training Project to BH (QNRC2016066) and YP (QNRC2016074).

## Conflict of Interest

The authors declare that the research was conducted in the absence of any commercial or financial relationships that could be construed as a potential conflict of interest.
