# Supplementary material for: Identification of Serum Exosomal hsa-circ-0004771 as a Novel Diagnostic Biomarker of Colorectal Cancer
Source: Front Genet. 2019 Nov 1;10:1096. doi: 10.3389/fgene.2019.01096 (PMC6838203; doi:10.3389/fgene.2019.01096)
Supplement: Supplementary file 1 [file Table_1.docx]

Supplement table 1. The top ten circRNAs with differential expression selected from GEO database and correlated data.

| **circRNAs** | **FC** | **logFC** | **P value** |
| --- | --- | --- | --- |
| hsa-circ-0001190 | 897.5 | 9.810 | <0.0001 |
| hsa-circ-0001136 | 1447.3 | 10.679 | <0.0001 |
| hsa-circ-0005823 | 1639.4 | 10.499 | <0.0001 |
| hsa-circ-0007694 | 521.1 | 9.025 | <0.0001 |
| hsa-circ-0004001 | 628.6 | 9.296 | <0.0001 |
| hsa-circ-0004771 | 18489.4 | 14.174 | <0.0001 |
| hsa-circ-0008521 | 249.2 | 7.961 | <0.0001 |
| hsa-circ-0005871 | 172.2 | 7.428 | <0.0001 |
| hsa-circ-0001062 | 3078.3 | 11.588 | <0.0001 |
| hsa-circ-0009076 | 622.5 | 9.282 | <0.0001 |
